# A Highly Linear CMOS Image Sensor Design Based on an Adaptive Nonlinear Ramp Generator and Fully Differential Pipeline Sampling Quantization with a Double Auto-Zeroing Technique

**DOI:** 10.3390/s20041046

**Published:** 2020-02-14

**Authors:** Chuangze Li, Benguang Han, Jie He, Zhongjie Guo, Longsheng Wu

**Affiliations:** 1 Xi’an Microelectronic Technology Institute, No. 198 Taibai South Road, Yanta District, Xi’an 710071, China; li_chuang_ze@163.com (C.L.); hejiexian@126.com (J.H.); 2 School of Microelectronics, Xi dian University, No. 2 Taibai South Road, Yanta District, Xi’an 710071, China; bghan@xidian.edu.cn; 3 Department of Electronic Engineering, Xi’an University of Technology, No. 5, Jinhua South Road, Beilin District, Xi’an 710054, China; zjguo@xaut.edu.cn

**Keywords:** CMOS image sensor, linearity, adaptive nonlinear ramp, fully differential pipeline, double auto-zeroing, high framerate, fixed pattern noise, floating diffusion, readout scheme, ramp generator circuit

## Abstract

For a complementary metal-oxide-semiconductor image sensor with highly linear, low noise and high frame rate, the nonlinear correction and frame rate improvement techniques are becoming very important. The in-pixel source follower transistor and the integration capacitor on the floating diffusion node cause linearity degradation. In order to address this problem, this paper proposes an adaptive nonlinear ramp generator circuit based on dummy pixels used in single-slope analog-to-digital converter topology for a complementary metal-oxide-semiconductor (CMOS) image sensor. In the proposed approach, the traditional linear ramp generator circuit is replaced with the new proposed adaptive nonlinear ramp generator circuit that can mitigate the nonlinearity of the pixel unit circuit, especially the gain nonlinearity of the source follower transistor and the integration capacitor nonlinearity of the floating diffusion node. Moreover, in order to enhance the frame rate and address the issue of high column fixed pattern noise, a new readout scheme of fully differential pipeline sampling quantization with a double auto-zeroing technique is proposed. Compared with the conventional readout structure without a fully differential pipeline sampling quantization technique and double auto-zeroing technique, the proposed readout scheme cannot only enhance the frame rate but can also improve the consistency of the offset and delay information of different column comparators and significantly reduce the column fixed pattern noise. The proposed techniques are simulated and verified with a prototype chip fabricated using typical 180 nm CMOS process technology. The obtained measurement results demonstrate that the overall nonlinearity of the CMOS image sensor is reduced from 1.03% to 0.047%, the efficiency of the comparator is improved from 85.3% to 100%, and the column fixed pattern noise is reduced from 0.43% to 0.019%.

## 1. Introduction

Highly linear complementary metal-oxide-semiconductor (CMOS) image sensors (CISs) have a wide range of applications in time-of-flight (ToF) ranging, medical imaging, space remote sensing imaging and scientific imaging [1,2,3,4,5,6]. The most critical element of a CMOS image sensor (CIS) is the column-parallel analog-to-digital converter (ADC). Various architectural designs of column-parallel ADCs have been proposed, including Cyclic-ADC, SAR-ADC, pipeline-ADC, single-slope ADC (SS_ADC) and multi-slope ADC [7,8,9,10,11,12]. The single-slope ADC structure is the most popular for column-parallel ADCs in CISs due to its simple circuit topologies and small layout area. The ramp generator circuit and the high-speed comparator in the SS-ADC structure are the most critical modules in determining the performance of the CIS.

Many research studies have been conducted to improve the linearity of CIS. According to the causes of nonlinearity of CISs, the solutions adopted in the literature are categorized into four types. The first type is employing the generated linear ramp signal buffered by the source follower (SF) transistor [8] to eliminate the gain nonlinearity of the SF transistor in pixel. However, the disadvantage of this method is that the nonlinearity of the integration capacitor (*C_FD_*) caused by the floating diffusion (FD) node still exists. The second type is to utilize the regulating output voltage follower method to calibrate the nonlinearity outside the pixel structure [2,3,13,14]. The third type is utilizing the unit-gain analog buffer instead of the SF in the pixel circuit [1,2,3,4] to reduce the gain nonlinearity of the SF transistor. However, this method increases the pixel area and reduces the fill factor. The fourth type is utilizing the off-chip high-precision ADC and digital-to-analog converter (DAC) to calibrate the nonlinearity of the CMOS image sensor system [1,2,3,15]. The advantages of this correction method are high linearity and high precision. However, the method is complicated and has a high cost.

The SS-ADC in the conventional CIS has different crossing point voltages and different offset voltages of the column-level comparator. In order to address this issue, a fully differential comparator with pipeline sampling quantization based on the double auto-zeroing (AZ) technique was proposed to improve the frame rate and reduce the column fixed pattern noise (FPN) in the spatial domain. In the literature [6], a classic CIS composed of a programmable gain amplifier (PGA) and an SS-ADC has been proposed for the column-level readout circuit. The advantage of this readout structure is that the dynamic range (DR) of the CIS can be extended, and the gain of the PGA can suppress the noises of the next stages (for example, the readout noise of the SS-ADC). However, the PGA itself has high noise, high power consumption, and the readout circuit sampling and quantization work in sequence without concurrent execution. Therefore, the comparator in the SS-ADC will have a fixed idle time, which reduces the efficiency of the comparator. The general readout scheme leads to the problem of large column FPN caused by different comparator crossing voltage levels. In order to address the above-mentioned problems, this paper proposes an adaptive nonlinear ramp generator circuit design and a readout circuit with fully differential pipeline sampling quantization based on the double AZ technique.

The remainder of this paper is organized as follows. Section 2 introduces the architecture of highly linear CIS. Section 3 describes the proposed adaptive nonlinear ramp generator technique and the fully differential pipeline sampling quantization based on the double AZ technique. The principle analysis of highly linear CIS with linear and nonlinear ramp generators is presented in Section 4. The simulation and experimental results of the fabricated CIS are discussed in Section 5, followed by conclusions in Section 6.

## 2. Image Sensor Architecture

**Figure 1:**
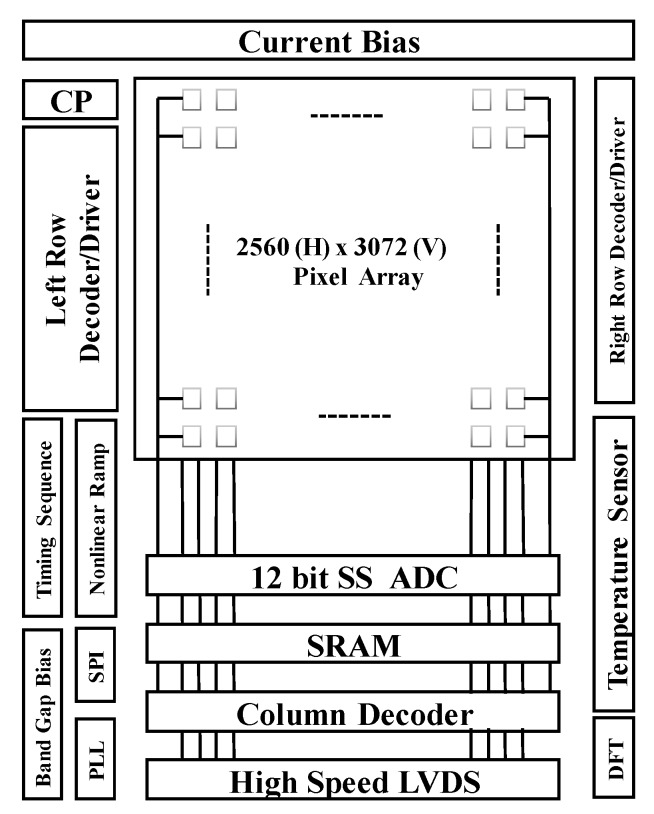
System architecture of the proposed highly linear complementary metal-oxide-semiconductor (CMOS) image sensor.

The CIS proposed in this paper consists of a typical 5T pixel [6] array, an adaptive nonlinear ramp generator, a readout circuit with fully differential pipeline sampling quantization based on the double AZ technique, row/column decoder and driver, timing sequence controller, phase-locked loop (PLL), charge pump, temperature sensor, and high-speed, high-precision, low-power LVDS serial data transfer circuit. The overall architecture of the proposed highly linear CIS is shown in Figure 1.

The central part of Figure 1 is a 2560 × 3072 active pixel array. The active pixel array is distributed around the dummy pixel. The dummy pixel can be used to generate an integration capacitor for an adaptive nonlinear ramp that is highly consistent with the characteristics of floating diffusion node capacitance. The left part of Figure 1 includes bandgap reference voltage generator circuit and current bias circuit, charge pump module, PLL module, timing sequence driving circuit, serial peripheral interface (SPI) and other modules. The right part of Figure 1 includes the temperature sensor, design for testability (DFT) circuit and the right row decoder driver circuit.

## 3. Proposed Techniques

The essential blocks of column-parallel SS-ADC in CIS is the ramp generator circuit and the high-speed readout circuit. This paper proposes an adaptive [16,17] nonlinear ramp generator and a fully differential pipeline sampling quantization scheme based on the double AZ technique. The two proposed techniques are dedicated for enhancing the performance of system linearity, improving the frame rate and reducing the column FPN of the CIS.

### 3.1. Nonlinear Ramp Generation Technique Based on Dummy Pixel Array

**Figure 2:**
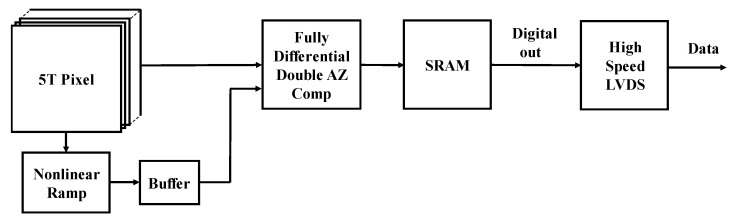
System signal process flow diagram of a highly linear CMOS image sensor based on nonlinear ramp generator.

Figure 2 shows the system signal process flow diagram of CIS using the nonlinear ramp generation technique based on the dummy pixel and fully differential pipeline sampling quantization technique.

**Figure 3:**
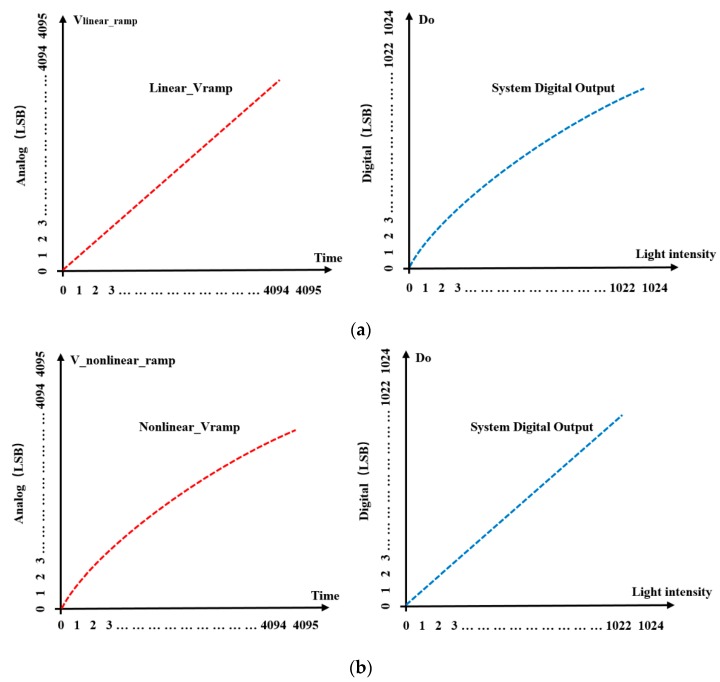
System digital number (DN) output values with linear and nonlinear ramp generators: (**a**) system DN output with linear ramp generator; (**b**) system DN output with a nonlinear ramp generator.

Figure 3a,b shows the system digital number (DN) outputs using the linear and the nonlinear ramp generators, respectively.

A typical SS-ADC utilizes the linear ramp generation technique, which cannot eliminate the nonlinearity of the pixel. The proposed nonlinear ramp generation technique based on dummy pixels can not only eliminate the gain nonlinearity of the SF, which changes with the input voltage but can also reduce the nonlinearity of *C_FD_* in the FD node.

**Figure 4:**
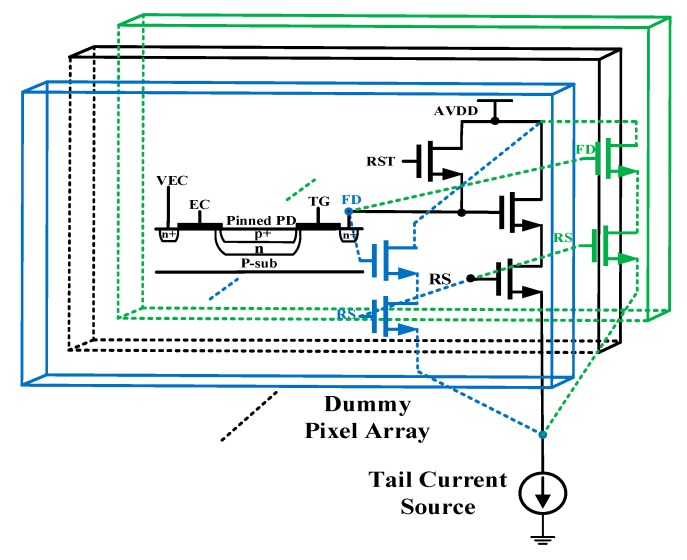
Schematic diagram illustrating the implementation of the integration capacitor by a floating diffusion node based on a dummy pixel array.

The proposed technique utilizes the multiple dummy pixel units surrounded by active pixel array, which are connected in parallel to form the *C_FD_* of the ramp generator circuit. That is, the equivalent *C_FD_* is generated by the parallel connection of multiple FD nodes of pixel units, and then a discrete sampling negative feedback technique is utilized to adaptively adjust the level of the current source. This technique produces an adaptively current source that creates a discharge path between the FD node’s capacitance and the ground, thereby generating a nonlinear ramp signal. Then the nonlinear signal is buffered by the SF transistor that has the same characteristics as the active pixel. The buffered ramp signal is then compared with the pixel output voltage by the fully differential pipeline sampling quantization comparator based on the double auto-zeroing technique. When the sampled active pixel output voltage signal *V_PIX_* is equal to the nonlinear ramp signal *V_ramp_*, the comparator toggles while the counter stops counting and stores the counter value into the static random-access memory (SRAM). Figure 4 shows the schematic diagram of a nonlinear ramp using the dummy pixel based on a typical voltage mode 5T pixel structure.

**Figure 5:**
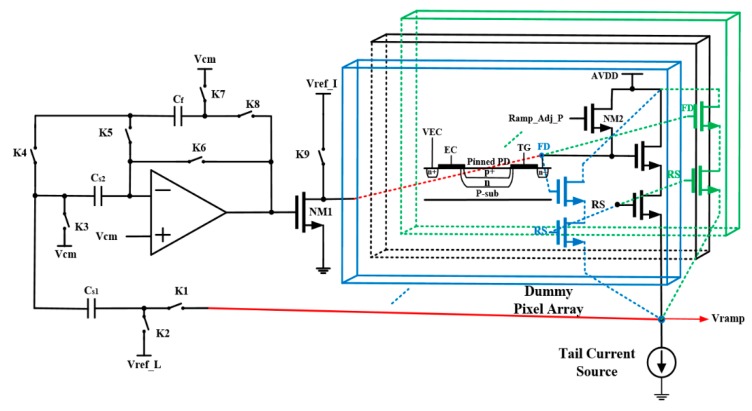
Schematic diagram of adaptive nonlinear ramp generator based on a dummy 5T pixel.

The schematic diagram of the adaptive nonlinear ramp generator based on the dummy pixel array is shown in Figure 5. The left part of Figure 5 shows the discrete negative feedback control circuit that generates the corresponding tail current source of nonlinear ramp generator. The right part of Figure 5 shows the circuit of *C_FD_* based on a dummy pixel. The *C_FD_* produced by the dummy pixel unit and the operational amplifier circuit constitutes the entire nonlinear ramp generator circuit, which is sampled and corrected by the adaptive discrete negative feedback control technique. The nonlinearity characteristic of the generated ramp signal is the same as the output of the pixel unit. Therefore, this method can reduce the nonlinearity caused by the capacitance of the FD node and SF’s variable gain in the pixel. Hence, the system linearity of the CIS is improved.

In the 5T pixel, the row selection transistor and the transfer gate transistor (including EC and TG) are connected to the power supply and analog ground, respectively, while the reset transistor NM2 is controlled by the timing signal (Ramp_Adj_P).

**Figure 6:**
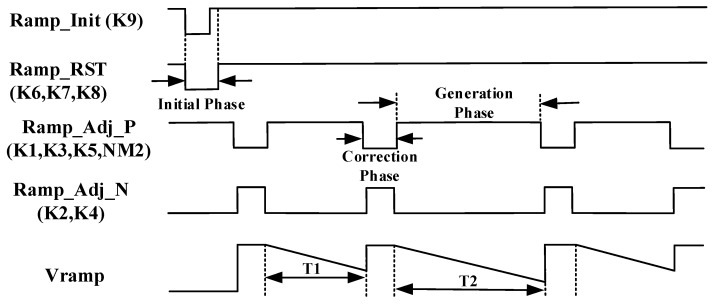
Timing diagram of adaptive nonlinear ramp generator.

The timing of the adaptive nonlinear ramp generator is divided into three phases. During the initial phase, both the pulse Ramp_Init (driving the switch K9) and the Ramp_RST (driving the switch K6–K9) are set low in the initial state of the adaptive nonlinear ramp generator circuit, while both are held high for other phases. The non-overlapped clocks Ramp_Adj_P and Ramp_Adj_N are driving the switches K1–K5 and NM2 to correct and generate the ramp, respectively. The second phase adaptively corrects the slope of the ramp signal. The third phase is the ramp generation phase. The timing diagram of the adaptive nonlinear ramp generator circuit is shown in Figure 6.

### 3.2. Fully Differential Pipeline Sampling Quantization Based on Double Auto-Zeroing Technique

Generally, the readout circuit for the CMOS image sensor consists of a programmable gain amplifier (PGA) and an SS-ADC. The sampling and the quantization phases are implemented by the PGA module and the SS-ADC, respectively. The sampling and the quantization phases usually work in sequential order. One disadvantage of this design used in the classic CIS is the inefficient use of the SS-ADC. Another disadvantage is that the comparator of SS-ADC has different crossing common-mode voltages that cause the offset and the delay information to vary in different columns, resulting in a large column FPN. In order to overcome the shortcomings of sampling and quantization phases, which cannot operate simultaneously for conventional SS-ADC, a readout circuit of fully differential pipeline sampling quantization based on a double auto-zeroing technique is proposed in this section. The proposed circuit realizes sampling and quantization executions concurrently to improve the frame rate and reduce the column FPN.

**Figure 7:**
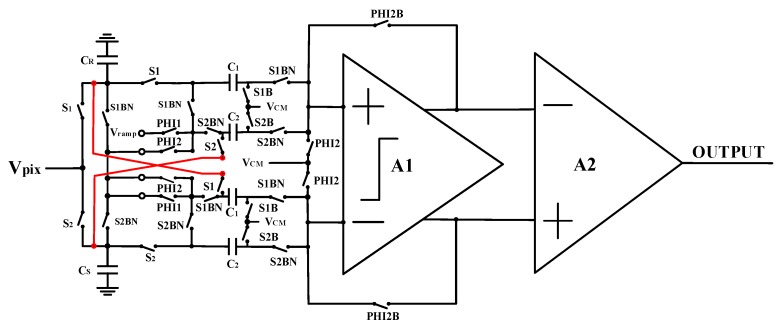
Fully differential comparator with pipeline sampling quantization based on the double auto-zeroing technique.

In the proposed design, a fully differential analog comparator with pipeline sampling quantization based on the double AZ technique is employed to improve the frame rate and reduce the influence of column FPN on the performance of CIS. Figure 7 shows the double AZ technique comparator circuit for fully differential pipeline sampling.

**Figure 8:**
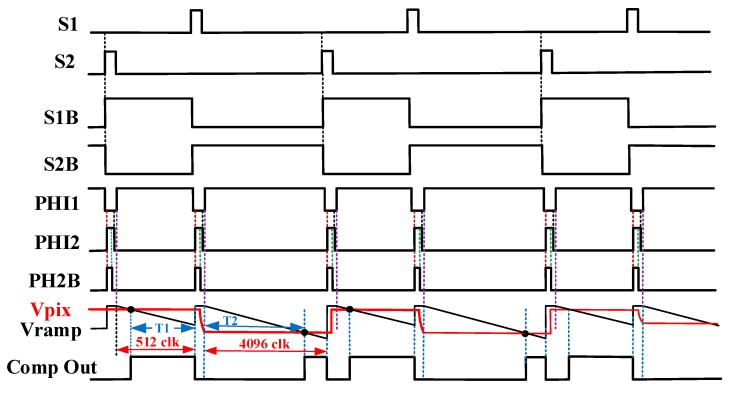
Working timing diagram of fully differential comparator with pipeline sampling quantization based on the double auto-zeroing technique.

In Figure 7, *C_R_* and *C_S_* are the reset and the signal sampling capacitors, respectively. S1 and S2 represent the sampling switch control signals in the signal and the reset phases, respectively. S1B, S1BN, and S2B, S2BN are the control switches related to the sampling reset and signal, respectively. Clock signal PHI1, PHI2 and PHI2B are utilized to realize double reset operation in the quantization phase. Where PHI2 and PHI2B are driven by a pair of non-overlapping clocks, and PHI2B is a control signal of the comparator in the reset phase ahead of PHI2 in phase by a constant time. Figure 8 shows a detailed working timing diagram for the fully differential comparator with pipeline sampling quantization based on the double AZ technique.

**Figure 9:**
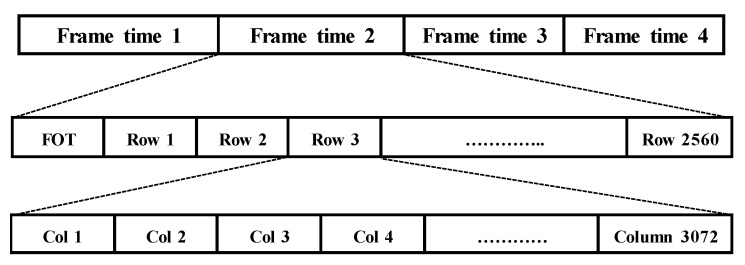
Readout timing of the column-parallel signal processing.

The column-parallel readout timing of the CIS is illustrated in Figure 9, and the whole frame time is defined as:

(1) $$T_{frame}=T_{FOT}+T_{row}\times N_{row}$$

where *T_FOT_* is the frame overhead time, *T_row_* is the row readout time, and *N_row_* is the number of rows of CIS. For the readout circuit structure of sequential sampling quantization, the time of row readout can be defined as:

(2) $$T_{row\_seq}=T_{sample}+\max(T_{ADC},T_{LVDS})$$

where *T_row_seq_* is the row sequential sampling time, *T_sample_* is the row sampling time, *T_ADC_* is the ADC conversion time and *T_LVDS_* is the LVDS output time. According to the column-parallel pipeline signal processing, the row readout time can be expressed as:

(3) $$T_{row}=\max(T_{sample},T_{ADC},T_{LVDS})$$

It can be observed from the above equations that the pipeline sampling structure will reduce the row readout time. Thus, the frame rate will be improved.

The proposed readout circuit combines the advantages of both pipeline sampling quantization and double AZ techniques. The proposed method saves two sampling times (the time of the correlated double sampling), avoids the waiting time as in the conventional readout scheme, increasing the efficiency of the comparator up to 100%, and improves the frame rate from 75 to 86 fps under the clock frequency of 400 MHz compared with the classic sequential sampling and quantization method. Since a double reset operation is performed in the quantization phase (double AZ technique), the values of signal and reset phases are all at the same constant crossing detector voltage level. Thus, the column FPN is significantly reduced and the inter-columns consistency of the CIS is effectively improved.

## 4. Analysis of Proposed Techniques

This section presents the analysis of the eliminating principle of nonlinearity when using the linear and the nonlinear ramp generators. Usually, the principle of a typical SS-ADC structure consisting of a high-speed comparator, a ramp generator circuit, and a digital counter is implemented by converting the counter time into the digital code, assuming that the photocurrent of the pinned photodiode is highly linear to the illumination intensity [1,2,3]. Equivalently, the analysis in this section uses time *t* to represent the digital output code of SS-ADC while conducting the relationship between the digital output code of an SS-ADC and the photocurrent when exploring both the linear and the nonlinear ramp generators.

### 4.1. Linearity Analysis of CMOS Image Sensor with Linear Ramp

Using a linear ramp, the ramp output voltage *V_ramp_* is defined as:

(4) $$V_{ramp}=\frac{I_{ramp}}{C}\times t$$

where *I_ramp_* is the discharging current of the ramp circuit, the integral capacitance *C* is a constant value, and *t* is the integration time of the ramp circuit. Since the illumination intensity changes linearly based on the above assumption, the output photocurrent of the pinned photodiode *I_pd_* is also changing linearly. When the voltage of the FD node voltage discharges for an integration time *T*_int_ with the photocurrent *I_pd_*, the resulting voltage *V_FD_* is defined as:

(5) $$V_{FD}=\frac{I_{pd}}{C_{FD}(V_{FD})}\times T_{\mathrm{int}}$$

where, *C_FD_*(*V_FD_*) is the total parasitic capacitance of the FD node, which is related to the voltage of the FD node, and is also one of the nonlinear causes of the pixel. The complete capacitance of the FD node is defined as [2,3]:

(6) $$C_{FD}=C_{RST\_OV}+C_{TX\_OV}+C_{SF\_OV}+C_{FD\_VERTICAL}+C_{METAL}$$

where, *C_METAL_*, *C_TX_OV_*, and *C_RST_OV_* are the parasitic capacitances related to the size of the metal wire, the TX transistor and the reset transistor, respectively. Once the sizes of the TX and the reset transistors in the pixel are determined, the corresponding parasitic capacitances will remain unchanged, while *C_FD_VERTICAL_*, and *C_SF_OV_* are related to the overlap parasitic capacitances of the floating diffusion node that changes with the voltage of the FD node. The output voltage of pixel *V_PIX_* is equal to the voltage of the floating diffusion node buffered by the SF, as shown below [2,3]:

(7) $$V_{PIX}=\frac{I_{pd}}{C_{FD}(V_{FD})}\times T_{\mathrm{int}}\times G_{SF}(V_{FD})$$

When the comparator of a single-slope ADC utilizes a linear ramp, the comparator toggles if the voltage *V_ramp_* is equal to *V_PIX_*, so depending on time *t*, Equation (5) can be obtained as:

(8) $$t=\frac{I_{pd}}{I_{ramp}}\times \frac{C}{C_{FD}(V_{FD})}\times G_{SF}(V_{FD})\times T_{\mathrm{int}}$$

where, *G_SF_*(*V_FD_*) is the gain of SF in the pixel. It can also be seen from Equation (8) that, due to the nonlinearity of integral capacitance of the FD node and the gain nonlinearity of the SF, nonlinearity exists between the integration time *t* and the photocurrent *I_pd_* when *T*_int_ is constant. That is, the integration time of the linear ramp generator has a nonlinear relationship with the integration time of the FD node in the pixel is constant (which can be equivalent to a linear change in the integration time when the illumination intensity is constant). It can be further observed that there is a nonlinear relationship between the digital output code of the SS-ADC and the linear ramp generator. Thus, the overall illumination intensity is not linear between the digital code (by representing the time *t*) of the SS-ADC and the illumination intensity *I_pd_*. The nonlinear relationship reduces the system linearity of the CMOS image sensor.

### 4.2. Linearity Analysis of CMOS Image Sensor with Adaptive Nonlinear Ramp

When using the adaptive nonlinear ramp proposed in this paper, the nonlinear ramp generator voltage is defined as:

(9) $$V_{ramp}=\frac{I_{ramp}}{K\times C_{FD}(V_{FD})}\times G_{SF}(V_{FD})\times t$$

where *K* is the scale factor, *I_ramp_* is the discharging current of the nonlinear ramp, and *C_FD_*(*V_FD_*) is the nonlinear capacitance produced by the dummy pixel, which is related to the voltage of the FD node. When the output voltage of pixel *V_PIX_* is equal to the ramp generator voltage *V_ramp_*, the output of the comparator is toggled, and the counter stops counting. At this moment, the output value of the digital counter represents the pixel signal. There is a linear relationship between the digital number (DN) and the ramp integration time *t* when using a nonlinear ramp. The integration time *t* is:

(10) $$t=K\times \frac{I_{pd}}{I_{ramp}}\times \frac{C_{\mathrm{FD}}(V_{ramp\_FD})}{C_{FD}(V_{FD})}\times \frac{G_{SF}(V_{ramp\_FD})}{G_{SF}(V_{FD})}\times T_{\mathrm{int}}$$

where *V_ramp_FD_* is the voltage of the FD node of the adaptive nonlinear ramp generator. According to Equation (10), when the voltage values *V_ramp_* and *V_PIX_* are equal, *C_FD_*(*V_ramp_FD_*), *C_FD_*(*V_FD_*), *G_SF_*(*V_ramp_FD_*) and *G_SF_*(*V_FD_*) are all also equal. Thus, the two variables in Equation (10) cancel each other out, thereby eliminating the nonlinear error caused by the FD node integral capacitance and the gain nonlinearity of the SF transistor. The linear relationship between the DN and the input derived from the above analysis is given as:

(11) $$t=K\times \frac{I_{pd}}{I_{ramp}}\times T_{\mathrm{int}}$$

where *K* is the number of dummy pixel units used for producing the nonlinear ramp integration capacitance.

## 5. Results

### 5.1. Simulation Results of Nonlinear Ramp Generation Circuit Based on Dummy Pixel

**Figure 10:**
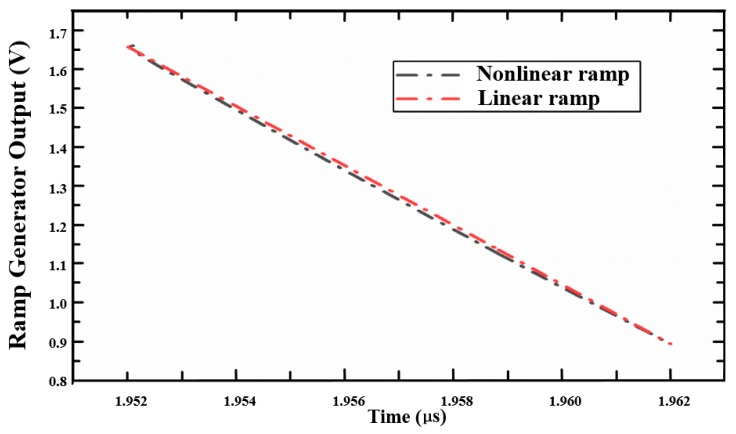
Comparison of nonlinear and linear ramp signals.

Figure 10 compares the simulation results of nonlinear and linear ramp generators based on the dummy pixel. The red and gray curves represent linear and nonlinear ramp signals, respectively. It can be seen from the simulation results that the nonlinear ramp signal already contains the nonlinear components of the pixel structure, which is the nonlinearity of the *C_FD_* of the FD node and the gain nonlinearity of the SF.

**Figure 11:**
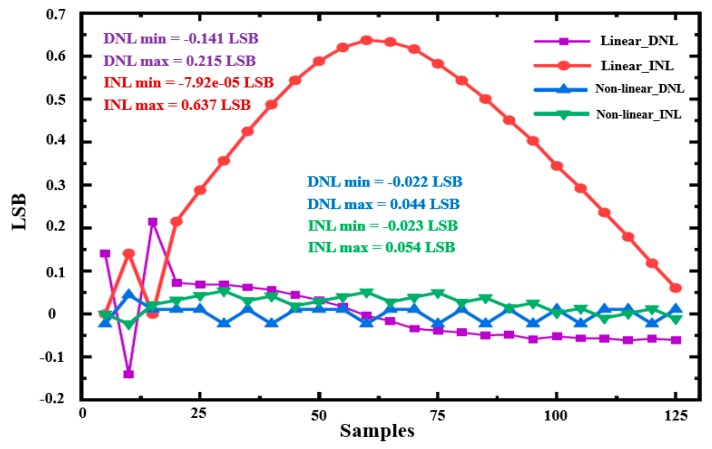
Simulation results of the linearity of CMOS image sensor (CIS) using the linear ramp and the adaptive nonlinear single-slope analog-to-digital converter (ADC) techniques.

Figure 11 shows the nonlinearity of CIS along with the linear change of photocurrent (*I_pd_*) when using the linear and the adaptive nonlinear ramp techniques. Figure 11 includes the nonlinearity characteristics of typical CIS using the linear and the adaptive nonlinear ramp techniques. It can be seen from the simulation results that the nonlinearity is significantly reduced between the output digital code and the photocurrent (light intensity). The maximum differential nonlinearity (DNL) is 0.044 LSB, and the maximum integral nonlinearity (INL) is 0.054 LSB.

### 5.2. Simulation Results of Fully Differential Pipeline Sampling Quantization with Double Auto-Zeroing Technique

**Figure 12:**
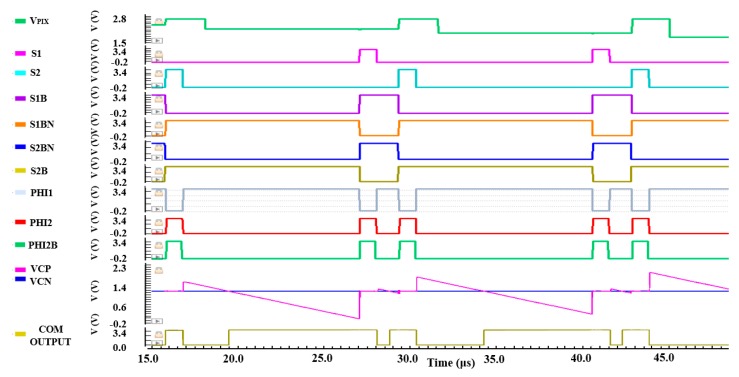
Operation timing of the fully differential pipeline sampling quantization with double auto-zeroing technique.

Figure 12 shows the simulation results of the operation timing of the fully differential comparator with pipeline sampling quantization based on the double AZ technique. It is easy to observe the correct function of the proposed fully differential comparator from the operation timing diagram. The crossing detector voltage level of the input positive level VCP is always the same as the negative level VCN for the comparator.

### 5.3. Experimental Result

**Figure 13:**
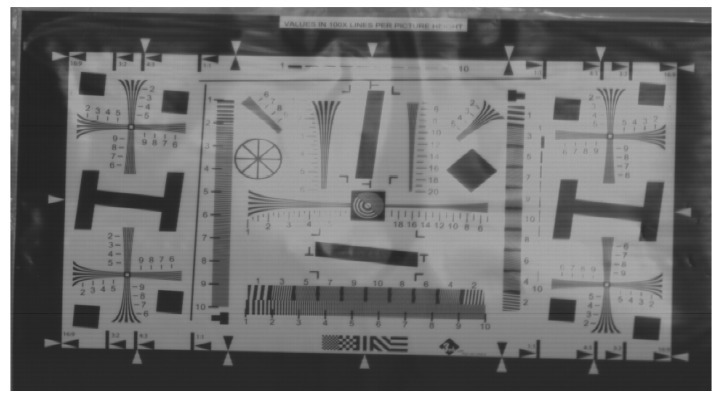
Sample image taken from the proposed highly linear CMOS image sensor.

The size of the pixel array in the proposed design is 2560 × 3072. Figure 13 shows the sample image taken by a typical global shutter pixel 5T structure with an adaptive nonlinear ramp generator and the fully differential comparator based on pipeline sampling quantization with the double AZ technique.

Table 1 compares the performance of the highly linear CMOS image sensor proposed in this paper with those reported in the literature.

## 6. Conclusions

In this paper, a novel adaptive nonlinear ramp generator design technique based on dummy pixels and a readout scheme of fully differential pipeline sampling quantization with a double auto-zeroing technique are proposed. The proposed ramp generator design can significantly improve the linearity of the pixel, which eliminates the nonlinearity caused by the capacitance of the floating diffusion node of the pixel and the gain nonlinearity of the SF transistor.

The proposed readout circuit improves the frame rate by 14% in the fully differential comparator with the pipeline sampling quantization technique, while increasing the efficiency of the comparator up to 100%. Moreover, the readout chain with the fully differential comparator based on the double auto-zeroing technique reduces the fixed pattern noise to 0.019%. A wide dynamic range of 81.3dB is also achieved by the proposed design without using the column amplifier gain stages. The resulting readout circuit noise is lowered to 7.9e-, and the system linearity is reduced to 0.047% using the adaptive nonlinear ramp technique.

## Figures and Tables

**Table 1:** Performance comparison of the proposed highly linear CMOS image sensor with the literature.

Reference	This Work	[2]	[3]	[3]	[8]
Process (nm)	180	180	180	180	40
Linearity improvement techniques	Adaptive nonlinear ramp based on dummy pixel	Pixel optimization and calibration	Analog buffer	Off-chip digital calibration	Linear ramp and source follower buffer
Array size	2560 × 3072	128 × 160	128 × 128	128 × 128	NA
Global/Rolling shutter	GS/RS	RS	RS	RS	RS
ADC Architecture	12/14 bit SS-ADC	12 bit SS-ADC	10 bit SS-ADC	10 bit SS-ADC	12 bit SS-ADC
Frame rate (fps)	86 (400 MHz clock)	NA	60 (12.5 MHz clock)	60	NA
Digital CDS	Y	Y	Y	Y	Y
Pixel size	6.5 µm × 6.5 µm	12 μm × 10 μm	10 μm × 10 μm	10 μm × 10 μm	NA
Fill factor	100% (BSI)	40% (FSI)	47% (FSI)	47% (FSI)	100% (3D BSI)
Pixel type	5T	CTIA	4T	4T	NA
Conversion gain	17.4 μV/e^−^	40 μV/e-	56.8 μV/e-	45.3 μV/e-	NA
Read noise	7.9 e^−^	16.4 (gain = 8)	4.12 (gain = 8)	4.17 (gain = 8)	261.5 μVrms
Full well capacity	91.7 ke^−^	30.613 ke^−^	17.27 ke^−^	20.96 ke^−^	NA
Dynamic range(dB)	81.3	65	72.4	74	71.8
Column FPN (%)	0.019	NA	NA	NA	0.028
SNR (dB)	49.4	44.2	42.1	42.9	NA
Nonlinearity (%)	0.047	0.095	0.058	0.06	NA
Dark current	8.3 pA/cm^2^@23 °C	NA	5.6pA/cm^2^	NA	NA
Per column Power	96.3 μW	NA	NA	NA	66.8 μW
